# Rethinking health systems in the context of urbanisation: challenges from four rapidly urbanising low-income and middle-income countries

**DOI:** 10.1136/bmjgh-2019-001501

**Published:** 2019-06-16

**Authors:** Helen Elsey, Irene Agyepong, Rumana Huque, Zahidul Quayyem, Sushil Baral, Bassey Ebenso, Chandani Kharel, Riffat Ara Shawon, Obinna Onwujekwe, Benjamin Uzochukwu, Justice Nonvignon, Genevieve Cecilia Aryeetey, Sumit Kane, Tim Ensor, Tolib Mirzoev

**Affiliations:** 1 Department of Health Sciences, University of York, York, North Yorkshire, UK; 2 Research and Development Division, Ghana Health Service, Accra, Greater Accra Region, Ghana; 3 Public Health Faculty, Ghana College of Physicians and Surgeons, Accra, Ghana; 4 Director, The ARK Foundation, Dhaka, Bangladesh; 5 Centre of Excellence for Urban Equity and Health, BRAC University James P Grant School of Public Health, Dhaka, Dhaka District, Bangladesh; 6 Research, HERDInternational, Kathmandu, Nepal; 7 Nuffield Centre for International Health and Development, University of Leeds, Leeds, UK; 8 Public Health Research, Centre for Injury Prevention and Research Bangladesh, Dhaka, Bangladesh; 9 Department of Pharmacology and Therapeutics, University of Nigeria Faculty of Medical Sciences, Nsukka, Enugu, Nigeria; 10 Health Policy Research Group, College of Medicine, Universiy of Nigeria, Enugu, Nigeria; 11 School of Public Health, University of Ghana, Legon, Greater Accra, Ghana; 12 Nossal Institute for Global Health Melbourne School of Population and Global Health, The University of Melbourne, Melbourne, Victoria, Australia; 13 Nuffield Centre for Health, University of Leeds, Leeds, UK; 14 Leeds Institute of Health Sciences, University of Leeds, Leeds, UK

**Keywords:** health systems, urban, non-communicable diseases, multi-sector, local government, nigeria, ghana, nepal, bangladesh, low- and middle- income country, urbanisation

## Abstract

The world is now predominantly urban; rapid and uncontrolled urbanisation continues across low-income and middle-income countries (LMICs). Health systems are struggling to respond to the challenges that urbanisation brings. While better-off urbanites can reap the benefits from the ‘urban advantage’, the poorest, particularly slum dwellers and the homeless, frequently experience worse health outcomes than their rural counterparts. In this position paper, we analyse the challenges urbanisation presents to health systems by drawing on examples from four LMICs: Nigeria, Ghana, Nepal and Bangladesh. Key challenges include: responding to the rising tide of non-communicable diseases and to the wider determinants of health, strengthening urban health governance to enable multisectoral responses, provision of accessible, quality primary healthcare and prevention from a plurality of providers. We consider how these challenges necessitate a rethink of our conceptualisation of health systems. We propose an urban health systems model that focuses on: multisectoral approaches that look beyond the health sector to act on the determinants of health; accountability to, and engagement with, urban residents through participatory decision making; and responses that recognise the plurality of health service providers. Within this model, we explicitly recognise the role of data and evidence to act as glue holding together this complex system and allowing incremental progress in equitable improvement in the health of urban populations.

**Summary box**

- Urban health systems must respond to rapid demographic, social and disease transition while also contending with a plurality of providers and a need to stimulate a multisectoral response to address the wider determinants of health.
- Rapid urbanisation presents challenges to traditional conceptualisation of health systems.
- Conceptualisation of urban health system must consider multisector responses, engagement with a plurality of providers, the role of local governments and engagement of urban residents, particularly the poor.
- Data and evidence, and technological advances in e-health, can provide the glue to hold together this complex urban health system.

## Introduction

Urbanisation continues at an ever-increasing rate across low-income and middle-income countries (LMICs), bringing with it changes to the disease burden and to the structural and intermediate determinants of health. Health inequalities in urban areas continue to grow; urban poor frequently experience worse health outcomes than their rural counterparts,^1^ yet the focus on urban health has not increased at a commensurate rate.^2^ In this paper, we highlight key challenges within urban contexts in LMICs that require us to rethink traditional ways of conceptualising the health system. We illustrate these key challenges with case studies from four countries, selected due to their differing points on the urbanisation journey—Bangladesh, Nepal, Ghana and Nigeria. The case studies are built on multiple sources of evidence including: (1) a rapid review of published evidence identified through searches of Global Health and Ovid databases for studies of urban health in the four countries. To gain a broad view, we prioritised systematic reviews, urban representative cross-sectional studies and qualitative research. (2) Government, non-governmental organisation (NGO), donor and media reports identified through searches of WHO/United Nations Human Settlements Programme (UN-HABITAT)/United Nations Development Programme (UNDP)/Unicef websites for each country; and (3) coauthors sharing of experiences of urban health; all coauthors are, in some form, engaged in health system policy making in their respective countries. As an analysis paper, we do not aim to present a systematic synthesis of the literature, and this paper should not be taken as such; instead we draw on this combination of published, unpublished and expert view to identify key urban health challenges. Summaries of the country case studies, with key citations, can be found in a online supplementary file. Drawing on these insights, we propose new ways to conceptualise health systems in urban areas, with a view to inform the development of interventions and policies to improve equity and health for all in urban areas.

10.1136/bmjgh-2019-001501.supp1 Supplementary data

## Health systems in a time rapid urbanisation

Recent work increasingly understands health systems as complex and adaptive with multiple relationships and interactions^3^ between elements of the system and the external context. These interactions create feedback loops that are dynamic with non-linear relationships between interventions and outcomes.^4^ Below, we show how urbanisation poses four key challenges that necessitate a conceptualisation of urban health systems that moves away from conceptualisations that emphasise top-down, monolithic healthcare structures.

### Challenge 1: responding to the rising tide of non-communicable diseases

Nowhere is the transition to non-communicable diseases (NCDs) more evident than in urban areas.^5–7^ Urbanisation itself has been identified as a determinant of health^8 9^ fuelling changes to intermediate determinants of health^10^ such as diet, exercise, tobacco and alcohol consumption behaviours that are driving the rise in NCDs, particularly among rural to urban migrants.^11^

Preventing and managing this growing burden of NCDs requires changes within the health sector, health workers require new skills to help patients change behaviours and self-manage their conditions, individual patient records are needed to manage patients over many years, referral and back-referral systems are needed for patients with complications, as well as drugs and diagnostic not previously on essential drug lists.^12 13^ For health services designed to deal with acute conditions and infectious diseases, such transitions present a major challenge.^7^

There is variation across our four case study countries in the level of readiness within healthcare services to manage patients with the four main NCDs: cardiovascular diseases, cancers, chronic respiratory diseases and diabetes, with Nigeria scoring only 3 out of 10 and Ghana scoring 9 out of 10 for the ‘general availability’ of essential NCD medicines.^14^ Preparedness to respond to mental health, a less prominent NCD but equally driven by urban stresses, is even more limited.^15–18^

### Challenge 2: responding to the wider determinants

The living conditions of urban areas, characterised by air,^19^ food^20^ and water pollution,^21^ lead to both NCDs and infectious disease. Water and sanitation were identified in all four countries as a key urban health challenge, with urban poor households and slum settlements both affected by poor quality and reliability of water provision and high numbers of households sharing toilets. Vector-borne diseases are a growing threat to urban life. Exponential growth in construction in booming megacities such as Dhaka, Bangladesh, has led to perfect breading grounds for *Aedes aegypti* mosquito, transmitting Zika, dengue and chikungunya. In Dhaka, 938 of 2599 constructions sites had infestations of Aedes.^22^ Understanding the true scale of such vector-borne disease is challenging due to the limited surveillance data.^23^ Such issues may be seen as beyond the remit of the health sector, and yet their impact on health and well-being is clearly evident.^24^

### Challenge 3: who is responsible for urban health? Role of local government and engagement with urban residents

As these wider structural and intermediate determinants sit outside the traditional remit of the healthcare system, there are inevitable challenges in identifying who should lead the response. Urban local governments are responsible for acting on these wider determinants through the provision and maintenance of transport systems, water and sanitation, planning and development. However, historically, local governments have been overlooked in funding and are under-resourced financially, and in terms of the skilled workforce, they are required to address complex, urban challenges.^25^ In Accra, Ghana, the local government budget per person per year is less than $50^26^ to respond to these wider determinants of health. Furthermore, pressure to address high profile and visible issues of priority to local elites such as traffic congestion and planning issues pushes general health issues such as mother and child health and NCDs down the priority list of local governments. This is typified with the response to dengue where, in Dhaka, local government has been criticised for visible actions such as spraying to appease public outcry, rather than prioritising more effective preventive measures.^22^

Where local government may be influenced by the voice of local elites and the media, poor urban residents were felt to have very limited involvement in identifying health priorities and solutions. The lack of inclusion of the urban poor in available data also undermines the possibility of local government response to address health inequities.^27^ Opportunities for improving responsiveness to the needs of the urban poor do exist though, as can be seen in Nepal’s new decentralised system federal system that emphasises bottom-up planning.

### Challenge 4: plurality of providers but limited free, quality primary health care

Our case study countries all have a well-defined rural health system, with a focus on primary care and often extensive cadres of community health workers and volunteers. The same structures rarely exist in cities, and attempts at their replication in the urban context lead to multiple challenges as seen in Ghana where the effective rural Community Health Planning and Services programme has faced multiple challenges in its adaptation to the urban environment communities, particularly: the need and request of urban residents for a greater range of services; challenges in sustaining and expanding engagement of communities and volunteer programmes in the context of transient communities working long hours who struggle to participate; and identifying mechanisms and resources to improve CHO motivation and skills through training and structures for career progression.^28^

Responsibility for the provision of primary care varies between the four case studies; unlike Ghana and Nigeria, in Bangladesh and Nepal, the responsibility for primary healthcare and prevention is devolved to local government, with no direct role for ministries of health. This level of devolution has led to some innovative responses to provision of primary care. In Bangladesh, responsibility for primary care is transferred to a public–private partnership, the Urban Primary Health Care Service Delivery Project (UPHCSDP) however, ensuring equitable coverage, continuity of care and referral systems is a major challenge and appropriate gatekeeping to limit the number of patients using tertiary care who could be better served in primary care. In Nepal, with the new federal structures, municipalities have increased decision making and budgetary powers to deliver healthcare to their populations, but coverage, quality and level of service provided remains a challenge.

In reality, with the growing urban population, ensuring quality and accessible primary care is a major challenge in all four countries. The urban poor can rarely access public health services due to their limited opening times during the long working day of poor daily wage earners. Instead they must rely on tertiary care or unregulated private providers; for example, in Bangladesh, 80% of health providers near slums were found to be private; the majority of whom were pharmacists or traditional doctors, only 37% with formal medical qualification.^29^

## Rethinking health systems models in a time of urbanisation

Our four case study sites highlight how the specific characteristics of rapid, uncontrolled urbanisation require a rethink of health systems models. Traditionally, the literature has conceptualised a health system as a monolithic entity, with interventions planned and managed in a top-down manner.^30^ This top-down approach is reflected in the structure of health services in rural areas with policies and interventions filtering down from the central Ministry of Health to the district hospital and then to primary care, often with the expectation of uniform effects across communities. The growing burden of the NCDs, the wider determinants of health, role of local governments, necessity of multisectoral responses and the plurality of health services present serious challenges to this traditional view of health systems.

Such critiques are becoming more vociferous; an UK International Development Committee (2014) critiqued UKAid and emphasised that: ‘Community services and public health are important parts of an effective and efficient health system. There can be a tendency, driven partly by standard health system models, to focus on curative care in formal national systems’^31^ (p. 24).

More recent health system frameworks have looked beyond the curative, healthcare system by drawing on the work of the Commission for Social Determinants of Health and suggested that the health and non-health sectors are equally important in the development of the health system at macro (policy), meso (subnational) and micro (interface with users/communities) level.^30^ Focusing on health outcomes through concepts such as ‘Health Action’ defined as any set of activities whose primary intent is to improve or maintain health.^32^ Further emphasis has been placed on health system ‘software’ such as ideas and interests, relations and power, and values and norms^33^ as well as ‘hardware’ identified in WHO’s building-blocks approach.

**Figure 1:**
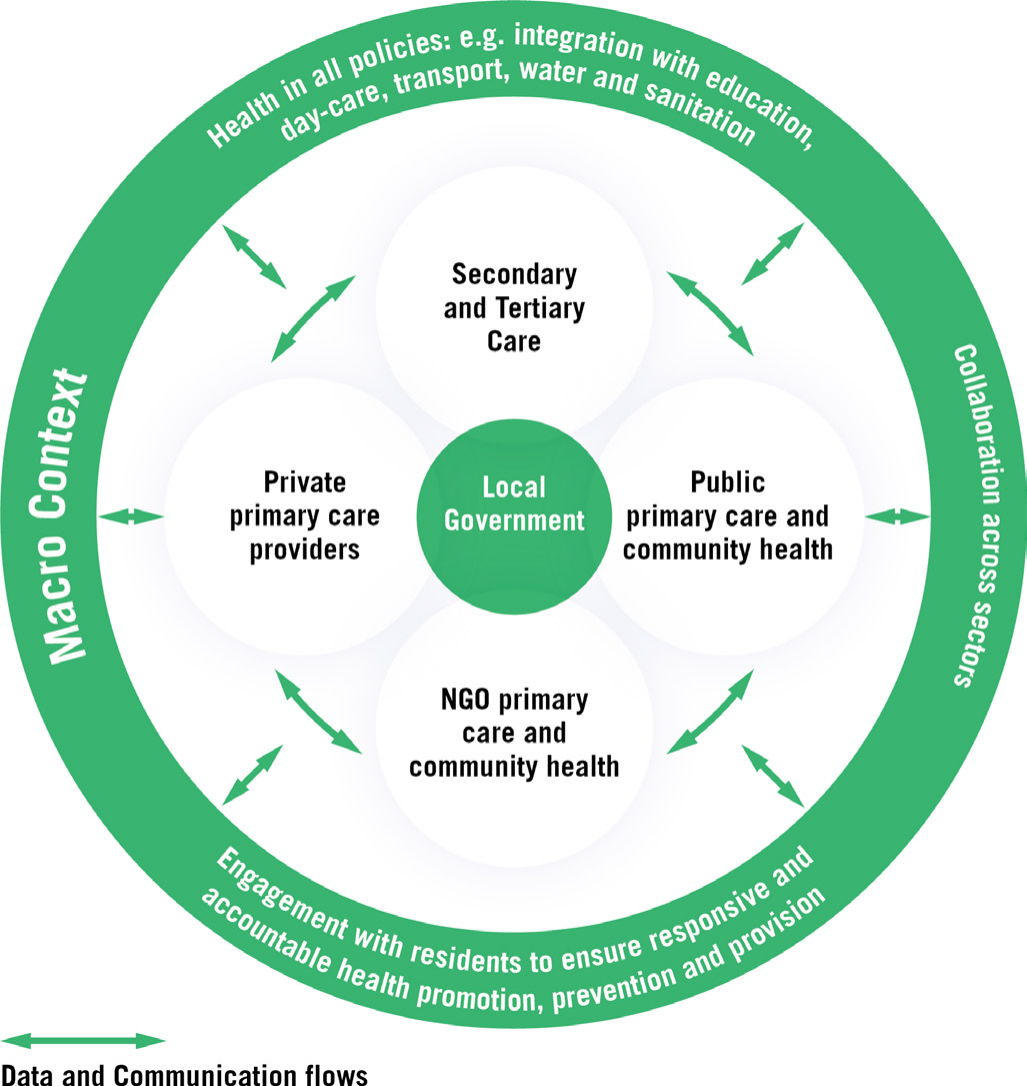
The urban health system.

Transparency, accountability and engagement of citizens at all levels are necessary to understand and tackle issues of, among other things, exclusion and discrimination in access to healthcare within households and communities. Frameworks for understanding urban health emphasise complexity and dynamism.^9 34 35^ This means that urban health outcomes are dependent on many interactions and unintended consequences are common.^36^Figure 1 below illustrates these concepts within the urban health system.

## Components of the complex urban health systems

Figure 1 illustrates the key components of the urban health system and their relationship with the macro urban context. This extended view of health systems focusing on multisectoral connexions, responsiveness to communities and recognition of the plurality of providers, liberates us in the response to improve urban health and achieve more equitable outcomes. Flows of information and communication between these different components of the urban health system are vital if the system is to function effectively.

### Multisectoral approach: the importance of looking beyond health

The inclusion of the non-health sector allows public health programmes to look for more creative ways of reaching urban poor communities. Exploring opportunities to co-opt non-health services into the response to health improvement and delivery of services provides many creative opportunities, for example, through schools,^37^ mosques,^38^ day-care centres^39^ as mechanisms for reaching the urban poor with messages of prevention, immunisation or nutrition campaigns or support for self-management. This reduces reliance on health workers giving health advice and attempting to stimulate individual behaviour change of the (proportionately few) patients that visit their health centres. Such an approach is vital to keep those seeking care to a manageable level, exhausting overstretched health services.

Local governments have potential to lead the multisectoral response to address wider determinants of health, being the champion for ‘Health in All Policies’.^40^ Donor partners and relevant ministries can play a valuable role in strengthening local government.

### Accountability to, and engagement with, urban residents

Engaging urban poor residents is vital if this complex health system is to be held accountable and responsive to health needs and address inequities. Commitment to establishing participatory decision-making processes is key to this, and there are examples within our case study countries where progress is being made. For example, the new constitution in Nepal allows for the strengthening of the role and function of Health Facility Operational Management Committees (HFOMCs) and local governments such as in Pokhara Municipality have taken this opportunity to strengthen bottom-up governance by facilitating HFOMCs attached to each urban primary healthcare clinic to identify vulnerable groups and individuals in their catchment areas and then identify ways of improving their access to services (S Baral, personal communication, 2018). Similarly, in Nigeria, a focus on local health decision making^41^ has led to the 2012–2020 strategy for Lagos and designated 376 ward health development committees as responsible for conducting needs assessments and planning, implementing solutions, mobilising human and material resources and monitoring and evaluating health activities.^42^

Disease transition also requires changes in how health professionals engage with urban residents, with a need to support patients to change behaviours and manage long-term conditions. Strengthening the software of the health system so that health professionals have the communication skills, attitudes and behaviours needed support patients in this way is now a priority.

Engaging urban residents in the process of developing population and community-wide prevention interventions is also vital in ensuring that campaigns and programmes are built on an understanding of the influence of urban living on risky behaviours such as tobacco and alcohol use, poor diet and limited physical activity. Recognising the diversity of the urban population and the changing gender and social norms due to urbanisation is key if health systems are to be responsive and ensure equity in health across urban areas.

### Recognition of the plurality of health service providers

The role played by the private sector in urban areas cannot be ignored. Despite the challenges in regulation and coordination, there is an increasing focus on finding ways to encourage private providers to improve quality and coverage, through social marketing, vouchers and contracting; however, evidence of cost-effectiveness is limited.^43^ Dhaka’s UPHCSDP model provides lessons for other contexts wishing to improve coordination between NGO, government and private sectors.

### Role of data and information

Data and information sharing can facilitate the coordination and integration needed across different sectors and providers ensuring responsiveness and accountability to urban residents. Examples exist, such as in Bangladesh, where data and the visualisation of services through platforms such as icddr,b’s ‘urban atlas’^44^ can provide an entry point for engaging with local government staff to understand and begin to monitor pluralistic health providers. The rapid spread of e-health systems can also help to strengthen the system linking pluralistic services, enabling management of long-term conditions through individual patient records and linking levels of care for referral and back-referral.^45 46^ Such linked data are a vital ingredient for the implementation research^47^ required to understand incremental improvements to the functioning of urban health systems.^36^

## Conclusions

Urbanisation requires us to rethink traditional views of health systems. Looking beyond the healthcare system and traditional views of a monolithic, controllable health system is vital. Increased focus is required on multisectoral approaches that look beyond the health sector to act on the determinants of health; accountability to, and engagement with, urban residents through participatory decision making; and recognition of the plurality of health service providers with greater emphasis and research on ways to improve quality and accessibility of healthcare across NGO, private and government facilities. Recognising the role of local government to act as a catalyst and mediator of a multisectoral responses while also engaging diverse urban communities is key. Data and evidence can act as glue, holding together this complex system and allowing evaluation of incremental progress in equitable improvement in the health of all those living in urban areas.
