# Supplementary material for: Rethinking health systems in the context of urbanisation: challenges from four rapidly urbanising low-income and middle-income countries
Source: BMJ Glob Health. 2019 Jun 16;4(3):e001501. doi: 10.1136/bmjgh-2019-001501 (PMC6577312; doi:10.1136/bmjgh-2019-001501)
Supplement: Supplementary data [file bmjgh-2019-001501supp001.docx]

**Supplementary File:** **Rethinking health systems in the context of urbanisation: challenges from four rapidly urbanising low- and middle- income countries.** Elsey, H. Agyepong, I., Huque, R., Quayyum, Z., Baral, S. Ebenso, B., Kharel, C., Riffat, A. S., Onwujekwe, O., Uzochukwu, B., Nonvignon, J., Aryeetey, G., Kane, S., Ensor, T., Mirzoev, T.

**Case Summaries: Key Issues in Urban Health in four rapidly urbanising countries: Ghana, Nigeria, Nepal and Bangladesh**

These case summaries provide supplementary information to our analysis paper ‘Rethinking health systems in the context of urbanisation’. The arguments presented in the paper are based on a rapid review of published and unpublished evidence and on the expert knowledge of our co-authors built on previous research studies, policy and programme work in the four case study countries, namely: Ghana, Nigeria, Nepal and Bangladesh.

Our rapid review focused on identifying published evidence through a search of Global Health and Ovid databases. The key words used within the search are provided in table 1. In selecting papers for detailed assessment, we prioritised systematic reviews, urban representative cross-sectional studies, qualitative research and well-informed opinion pieces. We identified relevant government, NGO, donor and media reports through searches of national government, WHO, UNHABITAT, UNDP and UNICEF websites for each country. We also searched newspaper and media websites for each of the four countries for relevant journalistic contributions. Finally, co-authors shared their experiences of working in urban health programming, research and policy. All co-authors are, in some form, engaged in health system research and the policy process in their respective countries. The four themes and conceptualisation of the urban health system presented in our paper emerged from discussions among the co-authors on challenges to equitable health for all urbanites, particularly the poorest. These themes are used to structure the country case studies presented below. It should be noted that this supplement is not meant as a systematic and comprehensive analysis of all urban health issues in the four countries. It is instead meant to provide readers with further references to follow-up on the issues identified in our paper.

Table 1: Search Terms used in Ovid and Global Health databases presented by theme

| Double burden of communicable and non-communicable disease | Wider determinants of health | Urban health governance, community engagement and multi-sectoral responses | Provision of primary care and prevention through a plurality of providers |
| --- | --- | --- | --- |
| urban and health  and NCDs/diabetes/heart disease/respiratory/ cancer/ stroke/mental health  and country name: Ghana, Nigeria, Nepal, Bangladesh | urban and health  and transport/pollution/ water/ sanitation/ gender/ equity/ poverty/ slum/ risk factor  and country name: Ghana, Nigeria, Nepal, Bangladesh | urban and health  and local/city government/municipality/ sector/governance/community engagement/civil society  and country name: Ghana, Nigeria, Nepal, Bangladesh | urban and health  and primary care/private/health provider/prevention  and country name: Ghana, Nigeria, Nepal, Bangladesh |

**1. Ghana**

**Non-communicable and communicable disease burden:** The continuing burden of communicable diseases particularly malaria, diarrhoea and increasing burden of HIV, coupled with the growing burden of non-communicable diseases, in particular mental health, cardiovascular diseases, hypertension, diabetes, obesity and cancers, were identified as an over-riding urban health challenge by co-authors in Ghana (IA, JN, GA). NCDs account for an estimated 43% of all deaths in Ghana (WHO NCD country profiles 2018 see Table 2 below). Urban specific data is limited however, Agyei-Mensah, S. et al. (2010) in their review of NCDs and CDs within Accra describe a "protracted" double burden of infectious and chronic with wealthy communities experiencing higher risk of chronic diseases and poor communities having a higher risk of infectious diseases as well as a double burden of NCDs and CDs. Systematic review evidence illustrates a higher prevalence of overweight (27.2% vs 16.7%) and obesity (20.6% vs 8.0%) among urban compared to rural dwellers*.*[1]

**Wider determinants of health**

Key wider determinants, as identified by co-authors and supported by published evidence, include the unplanned nature of urban areas resulting in inadequate infrastructure. In particular, poor sanitation, pollution and transport systems were identified as directly undermining health. The impacts are seen in the resulting disease burden of gastro-intestinal disease and respiratory conditions and the high burden of road traffic accidents resulting in deaths and life-changing disability. Poor transport system further impact on challenges with referral and response to emergencies. The published literature points to social as well as physical determinants, and while evidence is limited, social capital in urban slums appears to play a protective role in mental health with strong social networks buffering the negative repercussions of poor health and relative economic deprivation.[2] Poor waste management has been studied in Accra, finding only 13.5% of households served by door-to-door collection of solid waste, with the majority depositing waste at communal collection points, in open spaces and rivers, leading to significant associations with poor waste management and respiratory and gastro-intestinal health. [3]

**Urban health governance and multi-sectoral responses**

Poor regulation was a key issue identified by Ghanaian co-authors across the urban health-related systems, including: regulation of food, medicines including complementary and alternative medicines, private and NGO health providers. The lack of regulation and enforcement has led to an influx of counterfeit drugs, foods and beverages. We found no literature specifically addressing these issues in the urban context of Ghana. Challenges with the provision of safe water were better documented in the literature,[4] with attempts such as the SWITCH research process which used ‘Learning Alliances’ i.e. multi-stakeholder platforms of organisations active in the water sector, including government offices, NGOs and the private sector recommended as one way to instigate a multi-sectoral response. Authors identify resistance to this integrated approach however, as it requires organisations to look beyond their own mandates and barriers between institutions.[5]

**Primary care and prevention through a plurality of providers.** While urban communities may have greater physical access to a range of predominantly private clinics and pharmacies; access to quality, affordable services are limited, resulting in infant and child mortality five times higher in poor urban communities compared to the general urban population.[4] Ghana’s three-tier district health system has at its foundation the Community Health Planning and Services (CHPS) programme which has been successfully delivering universal access to health promotion, prevention and basic curative care in rural districts using community-based nurses known as Community Health Officers (CHOs) and volunteers.[6, 7] In study sites this has led to a reduction in childhood mortality by a third[8] and decline in total fertility by one birth.[9] However, despite government policy to scale-up CHPS nationally, implementation of CHPS in urban areas has been limited to a few pilot districts [6] and there are calls for more research to inform an urban CHPS model.[10] Pilot work has identified issues particular to urban areas: communities request a greater range of services, particularly to strengthen delivery of the Integrated Management of Childhood Illnesses (IMCI) approach, to sustain and expand engagement of communities and volunteer programmes and to improve CHO motivation and skills through training and structures for career progression.[10] Further issues identified by co-authors include over-reliance on vertical programmes, poor referral and emergency systems, including emergency transport and hospital emergency infrastructure were identified as co-authors are particular challenges. In terms of health financing there are challenges with rolling out health insurance scheme especially among vulnerable urban groups, affecting access to health services for all.

**2. Nigeria**

**Non-communicable and communicable disease burden:** Of the four countries, Nigeria has the lowest proportion of deaths due to NCDs, 43% (WHO NCD Country Profiles 2018, see table 2), however risk factors, have been increasing steadily, particularly obesity in women, rising from 5% in 2000 to a predicted 15% in 2025 and tobacco use in men being above global targets (WHO NCD Country Profiles 2018). Published evidence highlights the extent of the problem with hypertension and diabetes prevalence found to be 55.9% and 23.3% respectively in an urban community in north western Nigeria and women found to be at higher risk. [11] Risk factors are present across the social spectrum with government employees found to have low levels of physical activity and elevated risk for cardio-vascular disease.[12] The burden of communicable diseases especially malaria and HIV/AIDS persists. The need to reduce both disease burdens is captured in the 2018 National Strategic Health Development Plan and in the new National Health Policy of 2016.

**Wider determinants of health:** Co-authors emphasised the continuing trend of migration from rural areas to Nigerian cities in search of employment opportunities yet with increasing numbers of poor people living in urban areas, especially in the slums. One wider social determinant identified in the literature is violence, with it’s effects studied on young people in urban Nigeria, Kamndaya et al [13] found associations between multiple forms of violence experienced by young people and poor mental health outcomes in girls, and with poor health status in both girls and boys. While there are few studies in this area, an exploration of women’s working and living conditions in Lagos using survey data, identifies the poor socio-environmental conditions, inefficient transportation system, gender bias, work stress and poor quality of life as influencing poor nutrition, high levels of morbidity and repeated pregnancies all factors which can increase maternal mortality.[14] Poor environmental conditions were highlighted by co-authors and similarly a review of the literature identifies an urban health crises, with inadequate safe water supply and sanitation, poor solid waste management and inefficient, congested, and risky transport system. [15] Social determinants of health and lack of multi-sector approach in tackling many health problems in urban areas. In this case, the application of the ‘health-in-all policies’ principle in identifying and solving urban health problems is important, but unexplored in any systematic way. Older people are particularly at risk of catastrophic health expenditures, with 9.6% of elderly households at risk, particularly poor male-headed households where the man is main bread-winner. Interestingly, households with informal health financing arrangements were less likely to run into catastrophic expenditure [16]

**Urban health governance and multi-sectoral responses:** The need for coordination between sectors has been identified in the literature from several angles, including following an audit of 72 purposefully selected segments in Lagos which found a disordered built environment and transport infrastructure, with intermittent and inadequate power supply, waste management and poor governance. The audit highlights the need for interventions to leverage informal governance arrangements and build community stewardship.[17] This resonates with concerns from co-authors (OO, OB) on the very limited involvement of communities in identifying health priorities, both in terms of problems and solutions.

**Primary care and prevention through a plurality of providers:** Nigerian co-authors highlighted the numerous informal healthcare providers such as unregistered and registered patent medicine sellers, itinerant drug sellers, community health workers, recognising that they are the main sources of healthcare for the urban poor. The literature quantifies this issue e.g. Onyeonoro et al. [18] identify high use of patent medicine vendors (73%) in urban areas and these vendors were the most common sources of primary care following the onset of illness, while only 20% of the participants used formal care. Another cross-sectional study in urban South West Nigeria found the majority of the respondents rely on local doctors, spiritualists, and patent-medicine stores for health care. In addition, respondents' age, income, educational level and asset value were positively related to respondents' welfare status but household size, location, primary occupation and gender of respondents were negatively related [19]. These limitations in availability and accessibility of quality free primary care disproportionately affect the urban poor, and can be seen in high priority services such a maternal and child health services and many preventive services.

The National Health Policy of 2016 provides the policy direction for integration of informal providers into the formal PHC system, particularly under the strategic push in the country for Primary Health Care Under One Roof (PHCUOR). The recently implemented Basic Healthcare Provision Fund (BHCPF) and the scaling up of State Social Health Insurance Schemes targeted to achievement of UHC provide good opportunities for improving access to services for the urban poor. The opportunity includes the policy thrusts for improved control of non-communicable diseases at the PHC level.

**3. Nepal**

**Non-communicable and communicable disease burden:** Communicable diseases persist in urban areas of Nepal and the burden of non-communicable diseases has been increasing in Nepal [20], risk factors have been found to be particularly high in urban areas, e.g. Dhungana et al [21] in a cross-sectional survey of Kathmandu found smoking rates of 17.6%, alcohol consumption 29.4%, insufficient fruit and vegetables intake 98%, insufficient physical activity 21.0%, obesity 15.3%, hypertension 34.4%, diabetes 10.5% and high triglyceride levels 10.8%.[21] Young people in urban areas experience greater exposure to tobacco advertising than those in rural areas, with significant associations among young people starting to smoke (AOR [adjusted odds ratio] =2.49; 95% CI: 1.46-4.24).[22]

High levels of depression have been found in urban areas in Nepal, e.g. analysis of a nation wide cross-sectional study found positive associations with urban residence (AOR = 1.82; p < 0.001); [23] older people seem particularly badly affected in urban areas, e.g. rural 18.0%, [24] 60.6% (mild or above) in semi-urban areas [25]and in Kathmandu 47.3%.[26]

**Wider determinants of health:** Nepal’s Urban Health Policy 2072 (2015) [27] highlights the impact of urbanisation, industrialisation, unorganised settlements, rapid growth in migration, environmental pollution, changes in lifestyle and food habits in negatively impacting on the health of the urban population. Some of these impacts are quantified in the literature, e.g. the impact of lack of household water, limited opportunities form maintaining good hygiene, and insufficient time for child care were shown in a cross-sectional study to be associated with postnatal depression and physical health-related quality of life.[28] Severe air pollution has been identified in the Kathmandu valley with particular occupations affected, especially traffic police but also, indoor officer workers and urban residential area.[29] No adult childcare, so provision by other children and siblings was associated with an increased risk of malnutrition among children of urban working mothers. [51]

**Urban health governance and multi-sectoral responses**: the new context of federalism provides opportunities for municipalities to take the lead in bringing together the different sectors working at city level and enhancing bottom-up planning. [30] However, co-authors (SB, CK) emphasised the need to strengthen capacity of municipalities to take on their roles in planning and overseeing the delivery of quality health services and linking with sectors responsible for managing transport, environment, water and sanitation and development in order to address the wider determinants. This chimes with calls for a multi-sectoral integrated response to address the impacts of climate change in urban areas of Nepal, particularly the Kathmandu Valley. [31]

The literature provides some examples of multi-sectoral responses which are community-driven, such as the decentralized wastewater treatment systems (DEWATS) which was implemented in three communities in urban and peri-urban locations. Within these communities 43% of the population lacked access to a toilet, and virtually all wastewater and sewage is discharged into water bodies without treatment. Evaluation of the community-led DEWATS programme highlights the need to engage communities in urban health improvements and governance.[32]

**Primary care and prevention through a plurality of providers:** Co-authors emphasised how the strong rural health care structure is not replicated in urban areas, leaving a weak public primary care services and little option for the urban poor except to use private providers. This is quantified in the literature with a large cross-sectional survey finding a significant pro-rich distribution of general healthcare utilization in all service providers.[33] The government has been providing basic service from Community Urban Health Centres, however these are little used by the urban poor. [33] The Urban Health Policy 2072 (2015) seeks to improve access and use of quality services particularly by women, children, the poor and marginalised. Urban Health Centres (UHC) and Urban Health Promotion Centres (UHPC) are being strengthened and developed to increase access curative, preventive as well as promotive health services and public health programmes [27]

**4. Bangladesh**

**Non-communicable and communicable disease burden:** Bangladesh has the highest deaths due to NCDs (table 2 below) and much of the literature focuses on the burgeoning epidemics of CVD, diabetes, stroke and their risk factors, e.g. 30% of adults have metabolic syndrome.[34] Where studies are focused specifically on urban areas or comparisons with rural areas, prevalence has been found to be consistently higher in urban areas, e.g. the prevalence of type 2 diabetes and dyslipidemia were significantly higher in urban compared to rural populations (13.5% vs 6%, p<0.001; 41.5% vs 30%, p=0.007) respectively [35] and overweight and obesity.[36] Women are consistently more at risk of CVD, diabetes and stroke and risk factors. There is some evidence of rates and risk factors being particularly high in urban slums. Authors attribute this urban growth in NCDs to changes in lifestyle, particularly diet and physical activity patters fuelled by urbanisation.

Road traffic collisions in Bangladesh are an important cause of injuries in urban settings, particularly among men, those of working age, and vehicle drivers. [37] Vulnerable road users such as pedestrians and users of non-motorised vehicles are also at risk.

For many urban Bangladeshis, infectious diseases, particularly gastrointestinal diseases are still a major concern. Chowdhury’s cross-sectional study in Dhaka found an increased risk for diarrhoea among young children, males, those staying in rented houses, using non-sanitary toilets, transiency, living in an area with low education levels and limited use of safe drinking water source or living close to the hospital.[38] There appear to be increases in incidence during the seasonal epidemics of dengue fever and chikungunya and these two mosquito borne diseases have been identified by local government and the media as of particular concern to urban populations.[39] Urbanisation, with a plethora of construction sites and areas of stagnant water provides the perfect breading grounds for these vectors.[40]

**Wider determinants of health:** Co-authors from Bangladesh drew on recent qualitative research with urban poor communities [41] in addition to their own engagement with city corporation, ministry and donor networks (RH, SR, ZQ). Communities identified the stresses of urban living, particularly the inadequate transport system with excessive cars and resulting pollution coupled with broken and unsuitable roads and pavements making it hard to get about by foot. Densely populated homes and settlements with limited regular availability of gas, water and rubbish collection were seen as further stressors within the urban environment with many linking these stresses to poor mental health. Poor provision of clean water and sanitation is a key issue, with open drains which over-flow during the rainy season leading to damage of homes and increases in gastro-intestinal disease. These conditions are reflected in the literature and media reports, e.g. analysis of water and sanitation provision in urban areas in Bangladesh found that while access to water and sanitation services is overall quite high, issues of quality, safety, reliability, and liability were a particular challenge. Sharing of toilets is common with an average ratio 16 households to one facility and only 2% of household had access to safely managed sanitation.[42]

The interaction between social and environmental factors is also identified in literature, e.g. low levels of physical activity are found to be higher among women, and this is associated with these wider determinants such as a lack of neighbourhood safety, poor street lighting, lack of convenient places, unclean and untidy neighbourhood and poor weather as barriers to physical activity.[43]

Working patterns and conditions were associated with ill health and injuries in a number of studies, e.g. child labour was found to be positively and significantly associated with injury and illness with the intensity significantly higher in construction and manufacturing sectors than in other sectors.[44] The lack of appropriate childcare has been identified as a particular constraint to the health and development of under 5 children.[45]

**Urban health governance and multi-sectoral responses:** Co-authors emphasised the weakness of current urban planning structures and the need for multi-sectoral responses to deal with health problems and their wider determinants. Co-authors noted that health was only one of many areas addressed by City Corporations, and subsequently health was not given priority. Health issues, such as outbreaks of dengue fever, that capture the attention of all urbanites and the media were found to receive greater attention and resource than less visible health issues. [40] Work by Concern Worldwide Inc. (Concern) with two Bangladeshi municipal health departments[46] provides an valuable example of a successful attempt to build the capacity of local governments to bring sectors together to improve the delivery of maternal and child health preventive services. The process used systems thinking, facilitating local government actors using a Sustainability Framework to create a common vision and monitor progress. After 5 years the municipalities had a shared vision for sustainable health and were focused on equity-oriented services. They were able to strengthen Ward Health Committees and leverage resources between municipalities and the Ministry of Health. According to the evaluation, key structures and processes were maintained following elections and political changes.[46]

The literature identifies interesting potential interventions which reap synergies of multi-sectoral action such as green adaptations in the urban built environment such as rooftop gardens and urban agriculture which found high social acceptance (85%) and economic feasibility and was commonly practiced in Dhaka, particularly among house owners; however authors note that for other schemes such as pocket parks, green rooves, rainwater harvest, green facades/wall, porous pavement, and community garden which they were socially acceptable they would require greater community engagement and government leadership.[47]

**Primary care and prevention through a plurality of providers:** The plurality of providers in urban Bangladesh is evident and the extent of this has been quantified by several studies. For example, Adams et al [48] mapped health service providers in six slum areas in Dhaka and found over a thousand providers, 80% of which were private and the rest run by NGOs and the public sector. Of these private providers, 75% were pharmacies and non-formal or traditional doctors and 20% were consultation chambers. The limitations to quality are made clear by the finding that only 37% of private sector health staff held any kind of formal medical qualification.[48] Furthermore, the early closing time of many NGO and government clinics, 4-5pm, was found to limit their accessibility to the working poor. Co-authors further highlighted the limited level of awareness of poor urban households of the availability of free primary care at NGO and government clinics. The high use of private providers has been found to lead to financial hardship, as Uddin et al 2014 in their cross-sectional study of COPD patients found financial crises occurred significantly more frequently among those in urban compared to rural areas (12.5% vs. 2.4%).[49] They also found inadequate referral systems for both COPD and hypertension, with patients waiting until the disease severity increased. Similarly for diarrhoea, Chowdhury (2015) found that 80% of those with diarrhoea sought care initially from a non-professional healthcare provider.[38] There is recognition, both among co-authors and in the literature that the focus of the country’s health policies and programmes has traditionally been on rural areas, and this has undermined equitable access of the urban poor to quality health care. Govindaraj et al’s [50] mixed-methods assessment of urban health and nutrition services identified key system challenges including financing, regulation, service delivery, and public environmental health and advocates actions by policy makers and donors within and outside the health sector to address the issues among others that require policy attention.

**Table 2: Proportion of deaths due to non-communicable and communicable diseases**

|  | Ghana | Nigeria | Nepal | Bangladesh |
| --- | --- | --- | --- | --- |
| Cardiovascular diseases | 19% | 11% | 30% | 30% |
| Cancers | 5% | 4% | 9% | 12% |
| Chronic respiratory diseases | 2% | 2% | 10% | 10% |
| Diabetes | 3% | 1% | 4% | 3% |
| Injuries | 10% | 8% | 9% | 7% |
| Other NCDs | 13% | 12% | 13% | 12% |
| Communicable, maternal, perinatal and nutritional conditions | 48% | 63% | 25% | 26% |
| % of all deaths due to NCDs | **43%** | **29%** | **66%** | **67%** |

Source: WHO NCD Country Profiles 2018

**Table 3: Preparedness for non-communicable diseases**

|  | Ghana | Nigeria | Nepal | Bangladesh |
| --- | --- | --- | --- | --- |
| Proportion of population at high risk for CVD or with existing CVD (%) strokes (%) | no data | no data | no data | no data |
| Proportion of high risk persons receiving any drug therapy and counselling to prevent heart attacks and strokes (%) | no data | no data | no data | no data |
| Proportion of primary health care centres reported as offering CVD risk stratification 2017 | none | none | Less than 25% | none |
| Reported having CVD guidelines that are utilized in at least 50% of health facilities | yes | none | none | don't know |
| Number of essential NCD medicines reported as “generally available” 2017 | 9 out of 10 | 3 out of 10 | 7 out of 10 | 5 out of 10 |
| Number of essential NCD technologies reported as “generally available” | 5 out of 6 | 2 out of 6 | 5 out of 6 | 6 out of 6 |

Source: WHO NCD Country Profiles 2018

**References**

1. Ofori-Asenso R, Agyeman AA, Laar A. et al. Overweight and obesity epidemic in Ghana - a systematic review and meta-analysis. *BMC Public Health.* 2016, 16(1239).

2. Greif M and Nii-Amoo Dodoo F. How community physical, structural, and social stressors relate to mental health in the urban slums of Accra, Ghana. *Health & Place.* 2015, 33, pp.57-66.

3. Boadi K, Kuitunen M, Boadi K et al. Environmental and health impacts of household solid waste handling and disposal practices in third world cities: the case of the Accra Metropolitan Area, Ghana. *Journal of Environmental Health.* 2005, 68(4), pp.32-36.

4. Taylor P, Boussen C, Awunyo-Akaba J. et al. *Ghana Urban Health Assessment Environmental Health Project.* USAID, 2002.

5. Ibrahim S, Lubberding HJ, Drechsel P et al. Improving decision-making on interventions in the urban water system of Accra. (Special Issue: Water for urban agriculture.). *Urban Agriculture Magazine.* 2008, 20, pp.19-20.

6. Johnson FA, Frempong-Ainguah,F, Matthews Z et al. Evaluating the Impact of the Community-Based Health Planning and Services Initiative on Uptake of Skilled Birth Care in Ghana. *PLoS ONE.* 2015, 10(3), p.e0120556.

7. Phillips JF, Awoonor-Williams JK, Bawah A et al. What do you do with success? The science of scaling up a health systems strengthening intervention in Ghana. *BMC Health Services Research.* 2018, 18(1), p.484.

8. Pence B, Binka FN, Phillips JF, et al. The impact of the Navrongo Community Health and Family Planning Project on child mortality, 1993–2000. Paper presented Union for Population, Salvador, Brazil . In: *Union for Population Salvador, Brazil .* 2001.

9. Debpuur C, Phillips JF, Jackson, E. et al.. The impact of the Navrongo Project on contraceptive knowledge and use, reproductive preferences, and fertility. *Studies in Family Planning.* 2002, 33(141-63).

10. Nwameme AU, Tabong PT and Adongo PB. Implementing Community-based Health Planning and Services in impoverished urban communities: health workers' perspective. *BMC Health Serv Res.* 2018, 18(1), p.186.

11. Bello-Ovosi BO, Asuke S, Abdulrahman SO et al. Prevalence and correlates of hypertension and diabetes mellitus in an urban community in North-Western Nigeria. *Pan African Medical Journal.* 2018, 29(97).

12. Oyeyemi AL and Adeyemi O. Relationship of physical activity to cardiovascular risk factors in an urban population of Nigerian adults. *Archives of Public Health.* 2013, 71(6).

13. Kamndaya M, Pisa PT, Chersich MF et al. Intersections between polyvictimisation and mental health among adolescents in five urban disadvantaged settings: the role of gender. (Special Issue: Urban health at the edge: a series on reproductive health and HIV in inner-city Johannesburg.). *BMC Public Health.* 2017, 17(Suppl. 3).

14. Idowu AE, Edewor PA and Amoo EO. Working conditions and maternal health challenges in Lagos state, Nigeria. *Research on Humanities and Social Sciences.* 2014, 4(9), pp.136-147.

15. Aliyu AA and Amadu L. Urbanization, cities, and health: The challenges to Nigeria - A review. *Annals of African Medicine.* 2017, 16(4), pp.149-158.

16. Adisa O. Investigating determinants of catastrophic health spending among poorly insured elderly households in urban Nigeria. *International Journal for Equity in Health.* 2015, 14(79).

17. Mogo E, Litt J, Leiferman J et al. An audit of urban neighbourhoods in metropolitan Lagos, Nigeria. *Journal of Sustainable Development in Africa.* 2017, 19(1), pp.1-21.

18. Onyeonoro UU, Ogah OS, Ukegbu A et al. Urban-rural differences in health-care-seeking pattern of residents of Abia State, Nigeria, and the implication in the control of NCDs. *Health Services Insights.* 2016, 9, pp.29-36.

19. Oluwatayo IB. Healthcare service delivery system and households' welfare status in urban southwest Nigeria. *Journal of Human Ecology.* 2015, 50(2), pp.181-187.

20. Bishal G, Rajan S, Dinesh N et al. Prevalence of type 2 diabetes in Nepal: a systematic review and meta-analysis from 2000 to 2014. *Global Health Action.* 2015, 8(29088).

21. Dhungana RR, Thapa P, Devkota S et al. Prevalence of cardiovascular disease risk factors: A community-based cross-sectional study in a peri-urban community of Kathmandu, Nepal. *Indian Heart Journal.* 2018, 70(3), pp.S20-S27.

22. Aryal UR, Petzold M, Bondjers G et al. Correlates of smoking susceptibility among adolescents in a peri-urban area of Nepal: a population-based cross-sectional study in the Jhaukhel-Duwakot Health Demographic Surveillance Site. *Glob Health Action.* 2014, 7(24488).

23. Risal A, Manandhar K, Linde M et al. Anxiety and depression in Nepal: prevalence, comorbidity and associations. *Bmc Psychiatry.* 2016, 16(102), p.14.

24. Gupta AA, Lall AK, Das A et al. Health and Socioeconomic Status of the Elderly People Living in Hilly Areas of Pakhribas, Kosi Zone, Nepal. *Indian Journal of Community Medicine.* 2016, 41(4), pp.273-279.

25. Simkhada R, Wasti S P, Lee A C et al. Prevalence of depressive symptoms and its associated factors in older adults: a cross-sectional study in Kathmandu, Nepal. *Aging & Mental Health.* 2018, 22(6), pp.802-807.

26. Ranjan S, Bhattarai A and Dutta M.. Prevalence of depression among elderly people living in old age home in the capital city Kathmandu. *Health Renaissance.* 2014 11(3), p.6.

27. Primary Health Care Revitalisation Division. *Urban Health Policy 2072 - Unofficial translation,.* Kathmandu, Nepal, 2015.

28. Aihara Y, Shrestha S and Sharma J. Household water insecurity, depression and quality of life among postnatal women living in urban Nepal. *Journal of Water & Health.* 2016, 14(2), pp.317-324.

29. Gurung A and Bell ML Exposure to airborne particulate matter in Kathmandu Valley, Nepal. *Journal of Exposure Science & Environmental Epidemiology.* 2012, 22(3), pp.235-242.

30. Thapa R, Bam K, Tiwari P et al. Implementing Federalism in the Health System of Nepal: Opportunities and Challenges. *International Journal of Health Policy and Management.* 2019, 8(4), pp.195-198.

31. Marasini BR, Effects of climate change on urban health in the Kathmandu Valley. *Regional Health Forum.* 2010, 14(1), pp.15-18.

32. Bright-Davies L, Luthi C and Jachnow A DEWATS for urban Nepal: a comparative assessment for community wastewater management. *Waterlines.* 2015, 34(2), pp.119-138.

33. Saito E, Gilmour S, Yoneoka D et al. Inequality and inequity in healthcare utilization in urban Nepal: a cross-sectional observational study. *Health Policy and Planning.* 2016, 31(7), pp.817-824.

34. Chowdhury M, Anik AM, Farhana Z et al. Prevalence of metabolic syndrome in Bangladesh: a systematic review and meta-analysis of the studies. *BMC Public Health.* 2018, 18(308).

35. Kaniz F, Zwar NA, Milton AH et al. Prevalence of risk factors for cardiovascular diseases in Bangladesh: a systematic review and meta-analysis. *PLoS ONE.* 2016, 11(8).

36. Banik S and Rahman M. Prevalence of Overweight and Obesity in Bangladesh: a Systematic Review of the Literature. *Current Obesity Reports.* 2018, 7(4), pp.247-253.

37. Ahmad M, Rahman F, Haq M et al. (2015). Injuries among Drivers in RTA. *Bangladesh Journal of Medical Science,.* 2015, 14(4)(4), pp. 346-351.

38. Chowdhury F, Khan IA, Patel S et al. Diarrheal Illness and Healthcare Seeking Behavior among a Population at High Risk for Diarrhea in Dhaka, Bangladesh. *PLoS ONE.* 2015, 10(6), p.e0130105.

39. Dhaka Tribune. Dengue Prevalence Rising *Dhaka Tribune*. 2018.

40. The Guardian. Deadliest year for dengue fever in Bangladesh as cases explode in Dhaka. *The Guardian. London*. 2018.

41. Elsey H, Poudel AN, Ensor T et al. Improving household surveys and use of data to address health inequities in three Asian cities: protocol for the Surveys for Urban Equity (SUE) mixed methods and feasibility study. *BMJ Open.* 2018, 8(11), p.e024182.

42. Arias-Granada Y, Haque S, Joseph G et al. *Water and sanitation in Dhaka slums: access, quality, and informality in service provision.* Policy Research Working Paper - World Bank; 2018. (8552):47 pp. 26

43. Uddin R, Burton NW and Khan A. Perceived environmental barriers to physical activity in young adults in Dhaka City, Bangladesh - does gender matter? *International Health.* 2018, 10(1), pp.40-46.

44. Ahmed S and Ray R. Health consequences of child labour in Bangladesh. *Demographic Research.* 2014, 30,.

45. Das M, Elsey H, Shawon RA et al. Protocol to develop sustainable day care for children aged 1–4 years in disadvantaged urban communities in Dhaka, Bangladesh. *BMJ Open.* 2018, 8(7), p.e024101.

46. Sarriot EG, Kouletio M, Shamim J et al. Advancing the application of systems thinking in health: sustainability evaluation as learning and sense-making in a complex urban health system in Northern Bangladesh. *Health Research Policy and Systems.* 2014, 12(45).

47. Zinia NJ. and McShane P. Ecosystem services management: an evaluation of green adaptations for urban development in Dhaka, Bangladesh. *Landscape and Urban Planning.* 2018, 173, pp.23-32.

48. Adams AM, Islam R and Ahmed T. Who serves the urban poor? A geospatial and descriptive analysis of health services in slum settlements in Dhaka, Bangladesh. *Health Policy and Planning.* 2015, 30(suppl_1),

49. Uddin MJ, Nurul A, Haribondhu S et al. Consequences of hypertension and chronic obstructive pulmonary disease, healthcare-seeking behaviors of patients, and responses of the health system: a population-based cross-sectional study in Bangladesh. *BMC Public Health.* 2014, 14(547).

50. Ramesh G, Dhushyanth R, Secci F et al. *Health and nutrition in urban Bangladesh: social determinants and health sector governance. (Directions in development: Human Development).* Health and nutrition in urban Bangladesh: social determinants and health sector governance; 2018. xxi + 104 pp. 16 ref., 2018.

51. Nakahara S, Poudel K, Lopchan M, et al. 2006. Availability of childcare support and nutritional status of children of non-working and working mothers in urban Nepal. *American Journal of Human Biology*, 18, 169-181.
